# Literary Notes

**Published:** 1911-01

**Authors:** 


### LITERARY NOTES

The Fellows Company of New York, has issued a brochure entitled "Some Posological Hints and other Useful Information," which will prove a reminder on many minor points in practice often overlooked or forgotten. For example, classified under the head of absorption and elimination many suggestions are made as to methods of administration, and as to time required for a drug to take effect. Incompatibility is an important heading and a table of incompatibilities is appended. The table of overdose symptoms of drugs contains most valuable hints. The information contained in the booklet is gleaned from standard authorities on materia medica, therapeutics, practice, and from journalistic sources. It will be sent to physicians on application to The Fellows Company, 26 Christopher Street, New York.

Special Southern Number.—The January issue of the *American Journal of Surgery* will be composed entirely of original contributions from the pens of well known Southern Surgeons. Among these to appear may be mentioned: Pyuria, Howard A. Kelly, Baltimore; Transfusion of the Blood, its Indication and

Technic, J. Shelton Horsley, Richmond; Tumors of the Lower Jaw, The Form Most Frequently Found in the Negro, Willis F. Westmoreland, Atlanta; Pylorospasm, Stuart McGuire, Richmond; Prevention of Immediate Postoperative Pain by Quinine Injections, V. and V. W. Pleth, Seguin, Texas; The Importance of Educating the Public in Regard to Cancer, Southgate Leigh, Norfolk; Aerogenes Infections, George R. White, Richmond; Stricture of the Rectum, Complicating Fistulæ, C. S. Venable, San Antonio; Gastric Symptoms from a Surgical Viewpoint, Louis Frank, Louisville; Dr. Edgar D. Capps, Fort Worth, and H. Berlin, Chattanooga, will also contribute original articles to this number.

---

M. J. Breitenbach Company, New York, are sending out their Physicians Memorandum Books for 1911. Ours reached us just as the Journal for January is leaving the press. We stop it to say that this convenient desk reminder is a welcome visitor every year—never more than now. Send for it if you do not get it promptly.

